# Empowering teachers to screen, guide, and refer schoolchildren with behavioral and mental health problems. A pilot study to promote mental health in Iran

**DOI:** 10.3389/fpsyt.2022.894483

**Published:** 2022-08-04

**Authors:** Ramin Afshari, Mohammad Hossein Kaveh, Kamran Bagheri Lankarani, Damien Doolub, Nematollah Jaafari, Jeyran Ostovarfar

**Affiliations:** ^1^Research Center of Social Harms and Substance abuse, Shiraz University of Medical Sciences, Shiraz, Iran; ^2^Research Center for Health Sciences, Institute of Health, Shiraz University of Medical Sciences, Shiraz, Iran; ^3^Health Policy Research Center, Institute of Health, Shiraz University of Medical Sciences, Shiraz, Iran; ^4^Research Center on Cognition and Learning, CeRCA, CNRS UMR, University of Poitiers, University of Tours, Poitiers, France; ^5^Pierre Deniker Clinical Research Unit, Henri Laborit Hospital Centre, Poitiers, France; ^6^Faculty of Medicine and Pharmacy, University of Poitiers, Poitiers, France; ^7^Department of Health Promotion, School of Health, Shiraz University of Medical Sciences, Shiraz, Iran

**Keywords:** mental health, school-based program, teachers, school-age children, adolescents, strengths and difficulties questionnaire (SDQ)

## Abstract

**Background:**

Schools are ideal for promoting the mental health of school-age children, but the teachers' current knowledge is insufficient to play an influential role in mental health services at schools. Fortunately, however, teachers have a high sense of responsibility, interest, and talent to receive knowledge and the ability to participate in this field. This study aimed to examine whether a protocol focused on the role of teachers could identify, guide, and care for school-age children with behavioral and mental health problems.

**Method:**

The current research was a “before and after” pilot quasi-experimental study conducted in three elementary, secondary, and high schools. The main intervention consisted of a 5-h workshop based on a ready-to-use booklet for teachers conducted separately in each school. A total of 58 teachers and 872 school-age children were included using a judgmental sampling technique.

**Results:**

The pre-and post-workshop mean scores of teachers' knowledge about common mental disorders in school-age children were 6.21 ± 4.58 and 12.50 ± 7.27, respectively. According to the Strengths and Difficulties Questionnaire (SDQ), the teachers made 127 referrals, of whom 102 school-age children had problems. Consultants diagnosed 114 school children who reflected 90% of all teachers' recommendations needing psychological care and counseling. Finally, only 50 diagnosed school-age children were followed up and attended therapy sessions at counseling centers. The sensitivity and specificity of this brief intervention in detecting school-age children with psychological problems were respectively 80.3 and 98.0%.

**Conclusions:**

This study's results support teacher empowerment training's effectiveness in identifying and guiding school-age children needing mental health care. Psychological counseling programs in schools in various quantitative and qualitative dimensions, including responding to school-age children's needs and psychological problems, should be adequately evaluated, and appropriate measures should be taken to promote mental health services. Collaboration between health systems and the education department will increase the effectiveness of mental health programs' promotion and drug abuse prevention. These pilot data lead the way to designing scientifically sound follow-up studies that will concretely ascertain the benefit of this program.

## Introduction

Adolescence is a period of increased risk of developing mental health problems (1). Although there is a high prevalence of mental health problems among children and adolescents, recent research has shown that half of the mental health problems start at the age of fourteen and three-quarters at the age of 25 (1, 2). According to the literature, nearly 25% of children and adolescents worldwide suffer from psychiatric problems, epidemiological studies estimated their prevalence among adolescents between 8 and 20%, and Iran is not spared (3–5). According to Mohammadi et al. (2016), conduct disorders were the most significant mental health problems among 5,171 adolescents from five Iran provinces (6), and their findings were consistent with global reports on the high prevalence of mental health problems, especially among male adolescents rather than among females (5, 6).

Most, but not all, data support the hypothesis that mental health disorders and/or behavioral problems in early life are significant risk factors for developing substance use disorders (7). For instance, a 2018 cohort study reported that the risk of developing substance use disorders later in life was approximately 14.5 times higher for children and young people with mental disorders aged between 10 to 19 than their equals without these conditions (8). In addition, other studies have shown that only a small proportion of young people with mental health problems have used or received the services they needed. Moreover, fewer had appropriate care in specialized psychiatric institutions. For instance, according to Winstanleya and al., approximately 15% of adolescents have received behavioral therapies in the past year. More recently, in Australia, it was found that only 27–30% of adolescents with mental health disorders had received the relevant mental health services (9, 10).

In addition to health service centers, schools are optimal to incubate settings for mental health promotion plans for children and adolescents, as they are safe, cost-effective, and flexible places to offer a broad diversity of interventions (2, 11). The study of the school system's public, organizations, and societal infrastructure, resources, and capabilities appear to be particularly important in achieving the effectiveness and efficiency of these programs since school staff often takes on a highly influential role as children and adolescents compare themselves to them (12). In return, teenagers are less likely to engage in risky behaviors if they receive the attention they expect from their instructors (13).

Currently, and particularly in the education and mental health sectors, there is a demand to develop essential measures to create a natural process framework and effective partnerships among key stakeholders to achieve the goals of a sustainable school mental health promotion program, primarily to provide clear guidance on a process of care, including patient identification, referral, and follow-up (14, 15). It is essential to build capacity through appropriate training programs for this to work, including coordinating and empowering actors, particularly teachers, as frontline partners.

According to our observations and reports from the Ministry of Education executive staff, there was no integrated, coordinated program to identify, guide, care for, and follow up on school-age children needing mental health services (16). Contrariwise, only a small number of these school-age children (2.5% on average, estimated based on last year's unpublished statistics on the Counseling and Psychology Center of the Education Department) are accidentally identified at school (by teachers or counselors) and temporarily taken care of or taken from their families to non-school centers. Consequently, as part of an institutional substance abuse prevention program, this pilot study aimed to assess the impact of educating teachers on the identification, guidance, and care of school-age children with mental health and/or behavioral issues on their future trajectories.

## Methods

This pilot study with a “before and after” design was conducted in three schools - elementary, secondary (guidance), and high schools affiliated with the third department of education in Shiraz, the biggest city in southern Iran. Study units, i.e., schools, were chosen through a judgment sampling technique. First, the research team and representative persons from the education department collaboratively reviewed the available data and selected the schools that seemed more at risk of developing mental health and/or behavioral problems. Then all 58 teachers and 872 school-age children from these schools were recruited for the study project, in line with ethical processes^1^.

As a base for action, a protocol depicting the process of psychological care and services was developed, as shown in Table 1, where school teachers (ST), as the initial referral stage, are crucial frontline actors in identifying school-age children with mental health/behavioral problems. They likewise have an essential role, commensurate with their competence, in the ongoing care of sick students. School counselors/psychologists (SC), psychology and counseling centers affiliated with the education department (EDPCC), and the district's mental health services (DMHS) were considered respectively as the second to fourth referral levels.

**Table 1:** School-based Mental Health Promotion. Process of psychological care/services including steps and activities in terms of referral levels.

**Referral level**	**Activities/steps**	**Result**	**Follow-up action**
First School teacher (ST)	1. Carefully observe school-age children for screening signs of any behavioral problems as you were taught 2. Complete the SDQ for the student 3. Complete the referral form and refer the student to the SC	a. No sign detected b. Signs present	a. No action is needed b. Do steps 2 and 3 and follow instructions given by the SC
Second School counselor (SC)	4. Assess the referred student(s) 5. Document the process, inform the student's parent and take their consent 6. Fill the referral form and refer the student to DPCC	a. No problem b. Minor mental or behavioral problem exists c. Major mental or behavioral problem exists	a. Give feedback/instruction to the teacher b. Administer counseling/psychotherapy services + the above action (a) c. Do steps 5 and 6 and follow the instructions given by DPCC
Third Education Department's Psychology-Counseling Center (EDPCC)	7. Conduct advanced assessment and therapy as needed, including family therapy 8. Document the process, coordinate with the student's family 9. Fill the referral form and refer the student to DMHS	a. Improvement seen b. No significant improvement seen	a. Give feedback/instruction to the SC and/or the teacher b. Do steps 8 and 9 and follow the instructions given by DMHS
Fourth District's mental health services (DMHS)	10. Conduct advanced assessment and therapy as needed 11. Document the process	a. Improvement with continued outpatient care b. Decision with the participation of the family for hospitalization	a. Give feedback/instruction to the DPCC and/or SC and ST b. Critical decisions about changes in the student's family or school environment, if necessary

The main intervention was a 5-h workshop held separately for primary, secondary, and high school teachers. A booklet containing scientific facts about some common mental disorders/behavioral problems among children and adolescents written in a simple, understandable language was also offered to the teachers. The frequent conditions/problems were: depression, anxiety, bipolar disorder, bullying, attention-deficit hyperactivity disorder, oppositional defiant disorder, and conduct disorder.

Before that, research administrators conducted five meetings with senior managers in the education departments of the province and at the district level to gain their approval and support. Through a two-hour discussion session, counselors working in the selected schools and the counseling centers (DPCC) focused on the goal, objectives, care process, and expectations for implementing the project. In addition, an information session was held for the principals and teachers of each school. Lastly, an orientation session for parents was organized in the sampled schools to obtain their agreement and cooperation.

## Measurement tools

Teachers' knowledge of some common mental disorders/behavioral problems among children and adolescents was measured using a 28-item multiple-choice test. The correct and incorrect answers to each question in this test were scored 1 and 0, respectively. A panel of experts (*n* = 5) in psychology, psychiatry, and health education confirmed the content and face validity of the test. Its reliability was also confirmed by gauging a small study population (*n* = 25) and calculating the internal consistency coefficient (Cronbach α = 0.81).

The Strengths and Difficulties Questionnaire (SDQ) was used as a screening tool to identify emotional and behavioral problems in children and adolescents (17). It includes 25 positive and other negative items divided into five scales: conduct problems, hyperactivity-inattention, emotional symptoms, peer relationship problems, and pro-social behavior. An ordinal scale (0,−1, −2) is used to score each item of the SDQ. Scores of 0, 1, and 2 are assigned two positive items of 'Not True,' 'Somewhat True,' and 'Certainly True,' respectively.

The negative item's scores run opposite (i.e., 2,1 and 0). The scores of each of these scales, except those of social understanding, were added together to generate a total difficulty score. A zero and 40 reflect the minimum and maximum resultant scores, respectively (18). The study determined a cut-off score of 16 for a teacher form questionnaire used in this study based on the same score. In other words, each score falling in the category of normal or borderline was considered normal. Similar to the other relevant studies, the present study applied the exact cut-off as pointed out by three studies assessing and confirming the reliability and validity of the SDQ questionnaire in Iran (19–21).

Three referral forms were developed as part of this research project. The first was a referral form containing the main demographic characteristics of the student and a brief history, including school status and the commonly observed behavior problems. This form, attached to a completed SDQ, was used by the STs to refer needy school-age children to the SCs. The second form was developed for the SCs to refer school-age children needing advanced care to the EDPCC. In addition to the demographic situation, a more detailed history, the results of the psychological assessment, and the student's family situation obtained through interviews were provided. The third referral form was used by professional counselors working at EDPCC to refer needy school-age children to DMHS. It contains a more detailed description of the items mentioned above. In addition, the results and interpretations of other psychological tests used for school-age children were also provided.

Additionally, two forms were prepared to provide feedback from the higher referral to the lower reference level. The forms contain recommendations for monitoring the student's psychological care and tips for correcting performance. EPCC consultants and DMHC specialists used these forms. Finally, data were analyzed using SPSS^®^ version 28.0 for descriptive analysis.

## Results

On the survey concerning teachers' knowledge about the common mental disorders among school-age children, the pre-workshop and post-workshop mean scores and standard deviations were 6.21 ± 4.58 and 12.50 ± 7.27. There was a significant difference between the pre-and post-test scores on the Wilcoxon test (*p* < 0.001, *Z* = 4.43).

After completing the training sessions, teachers from the three schools identified 127 school-age children who needed psychological counseling out of the 872 schoolchildren. They came from primary, secondary, and high schools. After this first screening stage, teachers completed the Strengths and Difficulties Questionnaire (SDQ) and referred the schoolchildren to the school counselor. The school counselors carried out the scoring of the SDQ and interpretations of the test results. At the second referral level, counselors read and interpreted the SDQ results carefully and confirmed that 102 out of 127 school-age children required further assessment and possible mental health intervention. In other words, more than 80% of those school-age children referred by the teachers needed counseling and psychological care. In other words, teachers identified that nearly 90% of school-age children need counseling and psychological care (Table 2). As a comparison, according to the data obtained from the previous school year (without the program), the total number of school-age children in these three schools was 862. Of these, only 22 school-age children (2.5%) have been identified as needing psychological counseling services by school teachers or counselors.

**Table 2:** Frequency distribution of schoolchildren referred by school teachers to school counselor and their SDQ results.

		**The need for a referral**					**SDQ results**				
		**Yes**		**No**		**No problem detected**	**At least one problem detected**				
**Schools**	**Number of school-age children**	***N***	**%**	***N***	**%**	***N***	***N***	**Ratio considering the number of referrals in %**	**Ratio considering total school-age children in %**	**SE** **(%)**	**SP** **(%)**
Elementary school	208	50	24	158	76	7	43	86.0	20.7	86	95.6
Secondary school	260	50	19.2	210	80.8	5	35	70.0	13.5	70.0	97.6
High school	404	27	6.7	377	93.3	3	24	89.9	5.9	88.9	99.2
Total	872	127	14.6	745	85.4	15	102	80.3	11.7	80.3	98.0

SE, sensibility; SP: specificity; %: percentage; N: number; SDQ: Strengths and Difficulties Questionnaire.

Next, the counselors took a more in-depth look at all the school-age children referred by the teachers - including reviewing their personal stories, analyzing the questionnaire's subscales, and conducting clinical interviews. Compared to the results of the SDQ questionnaire, counselors diagnosed a higher proportion of school-age children needing to be referred to a counseling center (EDPCC third level referral) to receive adapted services (Table 3).

**Table 3:** Results of further mental assessment performed by school counselors on school-age children referred to them by school teachers.

**Schools**	**Number of school school-age children (a)**	**Number of teachers' referrals** **(b)**	**School-age children with at least one problem in SDQ (c)**	**Results of further assessment by school counselors**				
				**No problem found**		**Problem exists (referral is needed)**		
				***N* (d)**	***N* (e)**	**Ratio considering the number of referrals** **(e/b) in %**	**Ratio considering SDQ results (e/c) in %**	**Ratio considering total school-age children (e/a) in %**
Elementary school	**208**	**50**	**43**	**11**	**39**	78.0	90.7	18.8
Secondary school	260	50	35	1	49	98.0	140.0	18.9
High school	404	27	24	1	26	96.3	108.3	6.4
Total	872	127	102	13	114	89.8	118.8	13.1

Table 4 presents the frequency of school-age children who scored higher than the SDQ cut-off point - a teacher form scale. As can be seen, of the 127 school-age children referred, 102 (80.3%) were above the SDQ cut-off point. Most respondents scored higher in both hyperactivity (61.4%) and conduct (62.2%) scales. The percentage of referrals from elementary schools diagnosed with conduct problems was 72%, higher than in secondary and high schools.

**Table 4:** Frequency distribution of school-age children who achieved a score higher than the SDQ's cut-off point in each domain and types of school's form.

**Types of problems**	**Cut-off point**	**Elementary school**		**Secondary school**		**High school**		**Total**	
		***N***	**%**	***N***	**%**	***N***	**%**	***N***	**%**
Conduct problem	≥4	36	72.0	25	50.0	18	66.7	79	62.2
Hyperactivity	≥7	33	66.0	27	54.0	18	66.7	78	61.4
emotional problem	≥6	18	36.0	17	34.0	11	40.7	46	36.2
Peer problem	≥5	19	38.0	20	40.0	12	44.4	51	40.2
Social understanding	≤ 4	25	50.0	15	30.0	11	40.7	51	40.2
Total	≥16	43	86.0	35	70.0	24	88.9	102	80.3

Despite the investigators' follow-up to provide care to the 114 school-age children whom school counselors diagnosed during the second stage of the program (those who needed more counseling), only 50 of them were referred to counseling centers. They were received, and concerning the school population, the referrals, from the highest to the lowest, were respectively primary (9.6%), secondary (5.8%), and high school (3.7%; Table 5). Of all the referrals from school counselors addressed for psychological care at the counseling centers, only 51.3% of the elementary school school-age children, 30.6% of secondary school school-age children, and 57.7% of secondary school school-age children visited the service center.

**Table 5:** Number and proportion of school-age children in need of psychological care who were visited at the education department's psychological and counseling center (EDPCC).

**Schools**	**Number of school school-age children**	**Number of school-age children in need of care**				**Visit to get care at EDPCC**			
		**Based on teachers' referrals**	**Based on the SDQ**	**Based on** **the counselors' diagnosis**	**N**	**Ratio seeing the number of teachers' referrals**	**Ratio seeing SDQ results in %**	**Ratio seeing the counselors' diagnosis** **in %**	**Ratio seeing total school-age children in %**
Elementary school	208	50	43	39	20	40.0	46.5	51.3	9.6
Secondary school	260	50	35	49	15	30.0	42.9	30.6	5.8
High school	404	27	24	26	15	55.6	62.5	57.7	3.7
Total	872	127	102	114	50	39.4	49.0	43.9	5.7

Professional counselors at the counseling center of the education department provided 128 sessions for the 50 school-age children (i.e., an average of 2.56 sessions for each student) referred to them for assistance. The counselors held at least one and at most five sessions. Among the 50 referrals, four school-age children were identified as requiring medication. A psychiatric referral was made for three school-age children. The therapists recognized that 24 school-age children no longer needed the service and terminated the counseling program. However, it was decided to make more referrals for the other 26 school-age children to complete treatment.

## Discussion

Our study pre-test results showed that teachers' knowledge about school-age children's mental health problems/disorders was low. Mbwayo et al. found that elementary and secondary school teachers did not know enough to diagnose school-age children's psychological problems (22). However, compared with the workshop's pre-test, the post-test results showed the educational intervention's strength and success in increasing teachers' knowledge. Findings in a study by Kaveh et al. also showed a lack of knowledge in teachers about common psychological disorders among school-age children (23). They reported that the mean scores of the teacher's knowledge increased after the educational intervention in this population (23). Training and empowering teachers through appropriate training programs before and during service are essential factors that ensure the quality, adequacy, and persistence of mental health programs in schools where teachers play a crucial role (24).

In the present work, teachers identified 127 school-age children needing a referral to a school psychologist for further assessment and counseling after completing the training sessions and observable symptoms. Compared to the percentage of school-age children identified in the same schools last year (only 2.5%), the rate of school-age children identified by teachers as needing psychological services was significantly higher after teacher training in the present study. In the study of Kaveh et al., 106 school-age children were referred to the counselor by the teachers in the experimental group, of whom 79 persons were confirmed as having a disorder by the psychologist. The teachers in the control group referred only two school-age children to the counselors during the study (23). Desta et al. (2017) suggest a significant association between teacher training and more accurate recognition of children's problems (25). Furthermore, the SDQ questionnaire implementation has confirmed the problem's existence in more than 80% of school-age children.

Our findings showed that elementary and high school teachers displayed respectively the highest and lowest teacher participation in screening school-age children. The secondary school teachers' engagement was somewhere between elementary and high schools. In terms of gender, all secondary and high school teachers were male, but most teachers in primary school were female. Headley and Campbell (2011) reported that female teachers were more involved in screening children to counselors than male teachers (26). As the school staff is more likely to define an educational institution as a place where the primary function is to provide training or programs regarding education, not a health problem, health problems should be taken care of by the related professional (27).

According to the teachers' screening results, the implementation of the SDQ, and the school counselors performed on referred school-age children, about 13% of all school-age children needed psychological counseling services. Comparing this percentage (i.e., 13%) with the initial percentage of school-age children identified by teachers (i.e., 14.6%) indicates a relatively low false-positive rate. This portion is lower than the result of more exhaustive epidemiological studies in Iran. For instance, 17.8% of adolescents in a study by Rabani et al. obtained a more significant score than the cutoff point (28). Another study in Iran by Arman et al. showed that 26% of children and adolescents aged 6 to 18 years old in various districts of Isfahan had psychiatric problems. However, in Tehran, they found that the prevalence of children and adolescents with such issues was 25.8 and 13.7%, respectively (29). In another research conducted in Chile in the 4–18 years, older individuals reported a prevalence of 22.5% for all types of psychiatric disorders (30). According to their findings, 4–11 years old children had a higher rate of problems (27.8%) than 12–18 years old adolescents (16.5%). Given other relevant studies and the findings mentioned above, it seems that the data gathered from the elementary school referrals, in which a teacher stays in touch with his/her classroom school-age children for a more prolonged period, are more applicable to the real-world, the shorter their time spent with school-age children, the less time they allocated for screening. Furthermore, the lower proportion of cases observed in high schools can be attributed to the more significant number and variety of teachers.

After analyzing each completed SDQ factor separately, this study has reported that the conduct problem factor had the highest frequency (62.2%) among the teachers' referrals. The results were also similar when the analysis was based on different schools (19, 31). This is because a sample was taken from the target population in both studies and the surveying technique applied in the present study was not the same. In addition, the participants' selection was based only on the teachers' referrals.

According to our findings, the conduct problem was the only and most frequent problem among the school-age children surveyed through the SDQ by their teachers. Differences were observed in the prevalence of the conduct problem in the population, according to distinct types of diagnostic criteria, characteristics of the samples, age, and sex. According to community-based studies, the prevalence of conduct disorders in school-age children under age 18 ranged from 2 to 16% for males and 1.5 to 15.8% for females. There is much evidence that such disorders are more prevalent in boys than girls. However, community-based studies have not shown any gender difference in the lifetime prevalence of conduct disorders. The prevalence of conduct disorder in cities and suburbs (i.e., inner cities) was higher than in rural areas (6).

Also, conduct problems were more common in low-income families with low socioeconomic status. As described previously, the percentage of three factors, including social understanding (40.2%), peer relationship problems (40.2%), and emotional problems (36.2%), were approximately similar among all school-age children. However, the prevalence of disorders of social understanding (4%), peer problems (8%), and emotional problems (6%) were the same among Indian children (31). Also, the prevalence in Mashhad was based on the children's self-report and the SDQ parent form of social understanding (26.8%), peer problem (44.4%), and 15.9% with an emotional problem (32).

During the plan's second stage, when the school counselor recognized that 114 school-age children needed counseling services, only 50 (44%) were referred to the counseling center's therapists (Table 3). A study in Chile found that less than half of the children and adolescents in this country have been considered in need of mental health services, and most of them have not received these services.

The prevalence of hyperactivity disorder among the school-age children of the three schools, according to the SDQ screening in all children, was 8.9%. The estimate for the global prevalence of this disorder (5.2%) is more consistent with the prevalence in high schools (4.4%); (33). However, it is not easy to judge teachers' performance based on data from the counseling centers. Not all (114) school-age children were deemed necessary in the second stage of the referral process. The outcomes of the present study could be improved if a more comprehensive investigation of the current program was conducted at the outset of the school year. This could entail complete training programs for teachers and counselors, effective announcements to parents and raised knowledge, and strategic plans to improve the research project execution.

Designing a coherent protocol for organizing mental health promotion services in schools, including four referral levels, and explicitly defining their roles and tasks at each level and its empirical testing can be considered the strengths of the present study. However, the quasi-experimental design, lack of a control group, and lack of long-term follow-up should be regarded as the limitations of this study.

Furthermore, we also experienced several challenges in the implementation of this project. How these challenges are addressed may affect the outcome of a study. We, therefore, found it useful to address these challenges for future research. These can be categorized into pre-adoption, during-implementation, and follow-up. In the pre-adoption stage, getting cooperation and support from principals and teachers, ensuring parental involvement, and adherence to ethical principles, including providing a stigma-free, friendly mental health environment, are among the important challenges. In the third stage, follow-up, working together between the third and fourth levels is essential and must be managed effectively. Teacher empowerment facilitates project implementation if done before service. In addition, incentives can help increase teacher participation.

## Conclusion

Regarding the limited capacity of health systems in responding to the growing population in need of mental health care, the intervention designed in this study is proposed as a model for expanding and increasing the capacity of mental health service delivery systems in the community. This work supports the usefulness and importance of the cooperation between education and health sectors to increase access to mental health care services for the juvenile population. Despite some limitations, this study shows that teachers can influence school-based mental health promotion programs as long-term empowerment can make them more effective. Further research using experimental study plans and comparative studies considering different socio-economic and cultural environments must be carried out to standardize a globally appropriate protocol.

## Data availability statement

The raw data supporting the conclusions of this article will be made available by the authors, without undue reservation.

## Ethics statement

This study was approved by the Research Ethics Committee at Shiraz University of Medical Sciences (reference number HP73_89). Written informed consent to participate in this study was provided by the participants' legal guardian/next of kin.

## Author contributions

RA: writing–original draft and writing–review and editing. MK, KL, and JO: data curation, formal analysis, and investigation. MK, KL, JO, and NJ: conceptualization, funding acquisition methodology, project administration, resources, supervision, validation, writing–original draft, and writing–review and editing. KL, JO, and NJ: software and visualization. DD: validation, visualization, and writing–review and editing. All authors contributed to the article and approved the submitted version.

## Funding

This research project was financially supported by the Health Policy Research Centre affiliated with Shiraz University of Medical Sciences.

## Conflict of interest

The authors declare that the research was conducted in the absence of any commercial or financial relationships that could be construed as a potential conflict of interest.

## Publisher's note

All claims expressed in this article are solely those of the authors and do not necessarily represent those of their affiliated organizations, or those of the publisher, the editors and the reviewers. Any product that may be evaluated in this article, or claim that may be made by its manufacturer, is not guaranteed or endorsed by the publisher.

## Footnotes

^1^The authors assert that all procedures contributing to this work comply with the ethical standards of the relevant national and institutional committees on human experimentation and with the Helsinki Declaration of 1975, as revised in 2008.
